# Psychoanalysis and bioethics: a Lacanian approach to bioethical discourse

**DOI:** 10.1007/s11019-016-9698-1

**Published:** 2016-05-20

**Authors:** Hub Zwart

**Affiliations:** Department of Philosophy and Science Studies, Faculty of Science, Institute for Science, Innovation and Society (ISIS), Radboud University Nijmegen, P.O. Box 9010, 6500 GL Nijmegen, The Netherlands

**Keywords:** Bioethics, Lacanian psychoanalysis, Psychoanalysis and bioethics, Linguistics and bioethics, Ethics of animal research, Discourse analysis, Ethical expertise, Reproductive technologies, Forensic psychiatry

## Abstract

This article aims to develop a Lacanian approach to bioethics. Point of departure is the fact that both psychoanalysis and bioethics are practices of language, combining diagnostics with therapy. Subsequently, I will point out how Lacanian linguistics may help us to elucidate the dynamics of both psychoanalytical and bioethical discourse, using the movie One flew over the Cuckoo’s Nest and Sophocles’ tragedy Antigone as key examples. Next, I will explain the ‘topology’ of the bioethical landscape with the help of Lacan’s three dimensions: the imaginary, the symbolical and the real. This will culminate in an assessment of the dynamics of bioethical discourse with the help of Lacan’s theorem of the four discourses. Bioethics, I will argue, is not a homogeneous discourse. Rather, four modalities of bioethical discourse can be distinguished, all of them displaying specific weaknesses and strengths, opportunities and threats. This will be elucidated with the help of two case studies, namely the debates on human reproductive technologies and on the use of animals as biomedical research models.

## Introduction

In the opening lines of *Bioethics on the Couch*, the Norwegian bioethicist Jan Helge Solbakk explains how the idea for writing his paper came to him during a visit to the Freud Museum in Vienna (*Berggasse 19*). While relaxing on a replica of the famous couch, he sent “a message to my companion telling her where, in that particular moment, I was horizontally situated” (Solbakk 2013, p. 319). He immediately received a response, an injunction in fact, coming from the ‘Other’, as Lacan would phrase it, urging him to write a paper on bioethics and psychoanalysis; a summons with which he eventually complied (after the idea had been floating in his mind for some time), explaining how both practices have some very important features in common;—both tend to combine ‘analysis’ with ‘therapy’, for instance. But he also explained how, after modern medicine had ‘fathered’ bioethics, as he phrased it, the latter subsequently failed to reach the stage of autonomy, in Solbakk’s opinion at least, remaining too dependent on its parent, as “an integral part of the medico-scientific establishment”, a “handmaiden within the medico-industrial complex”, functioning as a “governance tool”, rather than as a truly critical voice (p. 320). In other words, bioethics could do with some self-analysis.

In this paper, I attempt something similar, adhering to a similar injunction, albeit more systematically than in Solbakk’s brief essay. My objective is to flesh out how (in my case: Lacanian) psychoanalysis may help us to unravel the intriguing dynamics and paradoxes of bioethical discourse, but also the uneasiness (or even discontent) which this discourse continues to invoke, among critics, but also among those who practice it, such as Solbakk. For indeed, experiences of ambivalence have accompanied bioethics from the very beginning, both internally and externally, so that the signifier ‘bioethicist’ refers to an exciting, but also controversial, perhaps even “impossible” profession, to use a phrase coined by Freud (1925/1948, p. 565, 1937/1950, p. 94). In other words, I intend to show how Lacanian psychoanalysis allows us to reflect on bioethics, not only as a success story, but also as a target of criticism and concern, even among authors who identify themselves academically with it. Its controversial status and reputation stems from the fact that bioethics is not a homogeneous discourse. Rather, four different modalities of bioethical discourse can be distinguished, all of them displaying specific weaknesses and strengths, opportunities and threats.

The idea for writing this paper has been fermenting for quite some time, ever since I became involved in bioethics (more than 25 years ago), as a continental philosopher for whom Lacanian psychoanalysis remained a source of inspiration. In other words, my endeavour amounts to self-analysis, an assessment ‘from within’, based on my experience as a *practicing* bioethicist: a participant in the discourse, using Lacan’s oeuvre as my frame of reference.

The design of my paper is as follows. After outlining what can be expected and gained from such an exercise, I will point out that both psychoanalysis and bioethics are practices of language in the radical sense. Both stress the primacy of discourse and the responsivity of moral subjects to the claims and imperatives of the Other. Therefore, the royal road towards bridging the gap or enacting a dialogue between psychoanalysis and bioethics runs via language, more specifically: via linguistics. Subsequently, I will explain the ‘topology’ of the bioethical landscape with the help of Lacan’s three dimensions: the imaginary, the symbolical and the real. Finally, the analysis will culminate in an assessment of the dynamics of bioethical discourse with the help of Lacan’s theorem of the four discourses, which will be elucidated by two exemplifications, namely the debates on human reproduction and on animal models in biomedical research.

## Preliminary exploration: psychoanalysis and bioethics

The objective of my paper may come as a surprise, as in mainstream bioethics Jacques Lacan is hardly ever mentioned. Even if we take into account that continental philosophy as such seems somewhat underrepresented (compared to analytic philosophy), prominent contemporaries such as Heidegger, Jaspers, Merleau-Ponty, Foucault, Bataille or even Deleuze have more presence in bioethics than Lacan. Moreover, bioethics does not figure prominently in Lacan’s oeuvre either, although, on closer inspection, highly interesting sections can be found on issues such as organ transplantation, genetics, molecular biology, embodiment and the cyborg-debate. But the point of my article is not to present a Lacanian view on specific issues (as I have done elsewhere), but rather to analyse the structure and dynamics of bioethical discourse *as such*.

Although the interaction between bioethics and Lacanianism so far has remained an idiosyncratic fold in the debate, on a more general level the dialogue between bioethics and psychoanalysis has been quite substantial over the years, oscillating between congeniality and animosity and back. Both represent a discourse as well as a practice in which assessments, diagnostics, interventions, interpretations, casuistry, etc. play an important role. Also, one could argue that, genealogically speaking, both bioethics and psychoanalysis build on discursive practices such as pastoral theology and the culture of confession, while both assist their clients in distinguishing right from wrong in complex and challenging situations. Moreover, as Solbakk phrases it: Freud’s “fingerprints” can be found on many bioethical terms, including the core concept of the autonomous Self (2013, p. 319), but this also goes for ‘technophobia’, ‘ambivalence’ and ‘denial’.

In addition, various aspects of psychoanalytical practice trigger analysis or even criticism from a bioethical viewpoint. An intriguing example is the case of Dora, the first extensive psychoanalytic case study published by Freud (1905/1942), who in his *Preface* admits to publishing this document (which contains a fair amount of intimate personal details concerning his former patient) without her consent, arguing that patients would never opt for psychoanalytic treatment if they suspected that confidentiality could thus be broken. Freud claimed that his duty as a scientist (to share his finding, so that future therapists and patients might profit from the insights gained) had to be given more weight than discretional duties towards single patients. And he took care to conceal Dora’s identity, notably by using a pseudonym, although her identity was nonetheless discovered (cf. Kochiras 2006). But again, my objective is not to reconstruct the actual history of mutual learning between bioethics and psychoanalysis, but rather to add an additional section, focussing on the profile of bioethics as a particular type of discourse. As indicated, the starting point of such a comparative analysis is language.

## Ethics and morality: the function of the signifier

Bioethics begins, I would argue, with individuals (moral subjects) finding themselves confronted with particular moral problem situations: biomedical dilemmas, for example, or instances of gross injustice: anything which arouses us from our usual free-floating indifference, forcing us to come up with a response (be it a comment, a judgment or a course of action).

Let me elucidate this with a concrete example. In 1975, while still a high school student, I saw the movie *One flew over the Cuckoo’s Nest*, set in a closed psychiatric hospital (Kesey 1962). Key protagonist Randle McMurphy is a delinquent who feigned insanity to avoid serving a custodial sentence and who largely lives by impulse, finding it impossible to comply with rules, schedules or discipline of any kind. He viscerally rejects authority and tries to violate, subvert or outwit anyone who confines him and his appetites in any way (Jennings 2010). But this time, he clearly underestimated the entangling power of the total institution he stumbled into: a psychiatric ward over which his nemesis, Nurse Mildred Ratched, exercises autocratic control. As the drama unfolds, a conflict seems inevitable, especially when McMurphy ferments resistance among the inmates, most of whom he believes to be sane people *made* insane by being kept in an insane place, to paraphrase Rosenhan (1973), as victims of Nurse Ratched’s stringent regime. When one of the inmates (a highly insecure young male, encouraged by McMurphy to have sex with a prostitute, for which he is subsequently reprimanded by Nurse Ratched) commits suicide, McMurphy physically attacks and almost strangles Ratched. As a result, he is lobotomised and surgically turned into a zombie, whereupon another inmate (a huge, Native American named Chief Bromden, who acts as the film’s narrator) makes his escape by tearing a heavy washbowl from the wall and throwing it through a window: an act of breaking-out, not only from the building as such, but also from the psychiatric logic it materialises.

I still vividly remember how the movie aroused in my peers and me an incongruent mixture of fairly strong moral responses. On the one hand, we clearly sympathised with McMurphy, who led the revolt against the politics of institutionalisation (a big issue in those days, when the anti-psychiatry movement was at its peak) and who, as a result, was involuntarily subjected to debilitating surgery, irreversibly altering his personality and quality of life (although I am sure we did not use any of these terms in our debate). On the other hand, some of us also sympathised with Nurse Ratched, who had fought desperately to survive, in a consistent effort to professionally manage an almost impossible situation, surrounded by (often intractable and potentially violent) males such as McMurphy: waiting for a moment of weakness on her part, or a professional mistake, anything that would provide them with an opportunity to strike.

The movie is very open to a psychoanalytic reading, moreover,—staging a case of ‘masculine protest’ against a powerful, intimidating woman, fuelled by castration anxiety and ending with lobotomy, as a stand-in for demasculinisation—, but let me focus on the bioethical dimension for now. Seen from this perspective, the movie enacts (in a highly dramatized, but nonetheless fairly convincing manner) a basic dilemma which torments penitential psychiatric practice up to this day, namely the tension between on the one hand the principle of autonomy, implying that invasive treatment is only legitimate if based on voluntary and informed consent (and beneficial to the patient) and, on the other hand, the risk of (severe) harm to others: McMurphy’s physical harassment of a senior professional, his *passage à l’acte*, as Lacan (who actually was a forensic psychiatrist himself) would phrase it.^1^

What is especially relevant, however, is that the moral responses invoked in subjects like me (not yet contaminated by years of professional philosophical training) are never purely visceral. They are never solely what ethologists would call a fight-flight-or-freeze response. Visual cues are important, no doubt, such as the Gestalt of the intimidating Nurse Ratched, with her omniscient, cynical gaze, contrasting sharply with the introvert silhouette of the Native American Chief, whose stoic equanimity suddenly gives way to his dramatic (and, to youngsters like me, quite inviting) gesture of escape. But such visual elements evoke responses which are nonetheless drenched in language: clad in and pre-structured by the culturally available moral labels and phrases currently in use, allowing us to literally come to terms with them. In other words, over and above mere physiological symptoms of anger or arousal, the movie (as a moral ‘stimulus’) inevitably triggers an assemblage of (often fragmentary) convictions, considerations and ideas, provisionally casted in moral terminology. Morality is not a matter of instinct, but permeated by language from the very outset.

And this is where ethics as a critical exercise comes in. How to transform muddled mixtures of ideas into a coherent and convincing analysis? How to articulate moral responses in such a way that they become consistent and sustainable? Socrates, the founding father of ethics, tried to do this by asking for moral reasons (*Why?* questions) and subsequently, as soon a particular concept (*x*) was brought in, by asking the (seemingly obvious) question *What is x?* If someone would argue, for instance, that McMurphy clearly had good reasons for physically harassing his foe, or that the psychiatrists clearly had good reasons for lobotomising their patient, Socrates would spur us to articulate our ‘reasons’ more explicitly, so as to probe and examine them. If someone argues, for instance, that an act of injustice had been committed, resulting in a case of suicide, or that McMurphy represented an otherwise uncontainable threat to other patients, to staff members, or even to the institution as a whole, Socrates would ask questions such as: *What is* justice? *What is* the purpose of a penitentiary psychiatric institution? *What is* proper treatment? Once questions of this kind are seriously addressed, the impromptu moral debate becomes sublated into ethical deliberation, and transposed from the realm of every-day morality into the intellectual arena of conceptual analysis. On this level, participants no longer simply state their viewpoints, their ‘way of seeing things’. Rather, they employ the calibrated vocabulary of ethics. The basic handiwork of professional ethics consists in analysing, clarifying and cleaning up a set of standardised terms, so as to facilitate systematic deliberation.^2^ Ethical expertise basically amounts to mastering a particular vocabulary, a jargon if you like, a ϰοινὴ or *lingua franca*, developed for the purpose of ethical diagnostics.

Or, to put it more explicitly in Lacanian terms: the ethical process starts with *imaginary* items, with a *Gestalt*; a tense empirical situation that triggers the imagination, involving a protagonist whose (more or less stereotypical) profile is bound to evoke specific reactions (of empathy or anger). In animal life, the imaginary register is the dominant one, but the human world is tainted by and saturated with language (Zwart 2014). Furthermore, Lacan would refer to the subsequent (interminable) handiwork of ethics as the ‘symbolisation’ of moral experience: replacing strong (provocative) images and phantasies by a network of ratified terms and principles (the ‘symbolic order’). Thus, bioethicists coin and refine the basic terms (‘signifiers’) which allow for a systematic analysis and assessment (‘domestication’) of subjective responses to complex moral problem situations.

Lacan elucidates this relationship between ethical terms and moral responses with the help of the algorithm (S/s), where uppercase (S) refers to the *signifiers* (the ethical terms produced and calibrated by professional ethicists) and lowercase (s) to the floating mass of experiences, emotions, convictions and considerations evoked by moral problem situations, emerging at the lower side of the bar (Lacan 1966a, p. 497). The former (the signifiers) are used to capture and domesticate the latter (the signified), while the *system* of signifiers (the terminological grid, the network of standardised, stabilised and formalised conceptions, emerging at the upper side of the bar) constitutes the *symbolic order*. This grid (this network of signifiers) will neither be seamless, nor fully consistent. There will always be something which we fail to articulate or grasp, invoking uneasiness, giving rise to symptoms of discontent in ethical discourse. Lacan refers to this unspeakable remainder, this annoying discursive recalcitrance as the Real, which notably reveals itself in moments of trauma, in moral tragedies for instance, when the gaps (the blind spots) in the symbolic network are suddenly (and quite painfully) laid bare.

To some extent, the imaginary is the realm of moral intuitions or moral sensitivity: the ability to discern the moral colouring of a situation, to have an eye for moral cues. The confrontation with concrete situations may evoke in us a range of positive or negative responses (from empathy and admiration up to repugnance, indignation and outrage). From a Lacanian perspective, however, our moral responses to acute problem situations are never purely spontaneous, purely ‘immediate’. Rather, morality provides a discursive ambiance, so that our responses are pre-structured by language (the discourse of the Other). And the subsequent process of *symbolisation* concurs with the iconoclastic tendency (discernible in scientific discourse in general, but in professional ethics as well) to replace images with words, and to gradually move from concrete situations (triggering intuitive moral responses) to more abstract and elaborate logical arguments (revolving around basic signifiers). In other words, symbolisation refers to the tendency to move from concrete *examples* (also in the sense of: exemplary individuals, idols, heroes, saints, villains, foes, etc.) towards standardised ethical terminology (for instance: sets of virtues or principles). Thus, the objective of bioethics, as an academic discipline, is to progress from the level of the particular (the visible and imaginable) towards the level of a professionally examined and calibrated terminology; from the level of narratives and parables towards the level of tested principles; and from striving, faltering and concretely situated *others* (Ratched, McMurphy, Bromden, etc.) to the symbolical order as the language of the *Other*. Thus, the network of signifiers (S) becomes increasingly able to articulate, structure and contain the floating mass of convictions, intuitions and ideas (s), notwithstanding the inherent tendency of the signified (s) to evade the signifier’s grasp (S). For indeed: moral experience keeps revealing the limited adequacy of accepted signifiers, keeps pointing to other (obfuscated) aspects, forcing ethicists to constantly adapt and refine their terminological grid. In other words, the relationship between signifier and signified remains highly precarious, for although we (as professional ethicists) may think that we ‘know’ what justice, autonomy, beneficence, etc. *is*, as soon as we are confronted with genuine moral dilemmas, a sense of embarrassment may again befall us. And although the signifiers provide a certain hold on the problem, offering moral guidance, the discord between our mastery of the ethical vocabulary on the one hand and the persisting uncertainty covered up by it on the other, is represented by the bar (/) separating (S) from (s): S/s. This bar represents a productive inhibition: we are continuously challenged but also frustrated in our efforts to make ethical discourse comply with our demands.

This distinction between the network of standard signifiers (S) and the floating mass of considerations, intuitions, convictions and ideas (s),—captured by the algorithm S/s—, corresponds to the distinction between *morality* (s) and *ethics* (S); or between “intuitive moral principles” and “critical thinking” (Hare 1981). A similar distinction is made in linguistics between actual, every-day language use (by the anonymous masses of native speakers: ‘morality’) and the formal rules and principles used to assess the quality and correctness of this language use (‘ethics’). Similar to ethics, linguistics is on the one hand a descriptive science (describing actual language use), but on the other hand a critical or even normative science, explaining how language *should* be used (in accordance with certified rules and principles).

An example of an ethicist using this analogy is John Rawls (1972/1980) who, referring to the work of Chomsky (1965), compares the sense of justice (which he presumes present in all “educated individuals”) to the “sense of grammaticalness” (i.e. the ability to produce or recognise well-formed sentences) of native language users, while comparing a full-fledged *theory* of justice to “explicit grammatical knowledge” (1972/1980, p. 47). Professional linguists aim to reconstruct the rules and principles which are unconscious and inaccessible to ordinary language users. This analogy provides another opportunity for furthering the bioethics-psychoanalysis dialogue because Lacan, in developing his S/s algorithm, likewise builds on insights borrowed from linguistics, notably the work of Ferdinand de Saussure (1957–1913), a contemporary of Freud, although, rather than straightforwardly copying the latter’s ideas, he adds some twists of his own (as we will see).

## Psychoanalysis, bioethics and linguistics

Lacan’s ambition was to reform psychoanalysis by connecting it with other twentieth-century research fields such as linguistics, ethology and computational logic. He intended to bring psychoanalysis on a par with modern science, notably through formalisation, thus enforcing what the (psychoanalytic) philosopher of science Bachelard (1938/1947) called an epistemological rupture: i.e. a leap from pre-scientific, intuitive forms of enquiry into systematic, stringent and formalised research (Lacan 1966a, p. 497).

In a famous essay entitled *The Agency of the Letter in the Unconscious*, Lacan (1966a) explicitly sets out to elucidate Freud’s work with the help of De Saussure’s linguistics (and vice versa). According to De Saussure (1916/1968), a linguistic sign (say: a word) is a bifacial phenomenon: an *association* of (on the one hand) an acoustic or literal unit (a series of sounds, a sequence of letters, an ideogram, etc.) and (on the other hand) a concept or idea. For the former, De Saussure suggests the term ‘signifier’ (*significant*), reserving the term ‘signified’ (*signifié*) for the latter (1916/1968, p. 99). Discourse is basically a sequence (a linear chain) of linguistic elements, representable by the following basic scheme (p. 159):

This linguistic system imposes itself on “speaking subjects” as an enormous mass of (collectively ratified) associations between signifier and signified, and De Saussure emphasises that speaking subjects are radically unable to challenge its tyranny, or to bring about significant changes in the functioning of the linguistic order (p. 101). In fact, speaking subjects are to a large extent “unconscious” of the rules that govern the language system and determine their speech and thoughts (p. 106).

Linguistic phenomena can be studied from two perspectives, moreover, namely *synchronically* (studying language as a system, at a particular historical moment in time, for instance: the present) and *diachronically* (studying the ways in which language systems evolve over time, focussing on what De Saussure refers to as linguistic ‘displacements’ or events).

Linguistic changes occur continuously. Basically, they are *displacements* (‘déplacements’) in the *association* (relationship, ‘rapport’) between signifier and signified. A signifier itself may change (a particular letter or sound may be substituted, or dropped, for instance) or a signifier may become detached from a particular signified and reconnected with another. But again, speaking subjects are usually unaware of such alternations, occurring “subconsciously” (p. 163, p. 171). Linguistic idiosyncrasies (such as: exceptions to the rules of grammar) are usually “symptoms” (p. 232) of previous displacements which occurred somewhere in the past, but again, as a rule speaking subjects are hardly conscious of the diachronic dimension of their language at all (which is usually “repressed”).

Language, moreover, is basically a system of (discrete) *differences* (between sounds, letters, characters, etc.), allowing for differentiation. As an example, De Saussure mentions the words ‘father’ and ‘mother’ (*père* and *mère* in French, p. 167), where a difference of just one consonant corresponds with the difference in gender. Thus, the absence or presence of a particular “member” or “articulus” (p. 156) allows for a specific connection between signifier and signified.

Lacan’s aim of merging psychoanalysis with linguistics (Freud with De Saussure) is evidently facilitated by the fact that the latter uses so many terms which are part of the psychoanalytic vocabulary as well;—such as ‘association’ (*Assoziation*), ‘displacement’ (*Verschiebung*), ‘unconscious’ (*Unbewusst*), ‘resistance’, *rapport*, the ‘speaking subject’, etc. For Lacan, this points to a more basic congruence between both fields. De Saussure’s work, he argues, allows us to articulate more precisely what psychoanalysis really is about. It is unfortunate that *The Interpretation of Dreams* was published 16 years before De Saussure’s *Course in General Linguistics* appeared in print (1966a, b, p. 513). Had Freud known De Saussure’s linguistic theories, Lacan surmises, he undoubtedly would have used them, not only in view of his keen interest in linguistic phenomena,—such as slips of the tongue (Freud 1917/1940), language jokes (1905/1940), etymology (1910/1943) and, of course, the role of letters, words, phrases, cryptograms, rebuses and other literal elements in dreams, as the “royal road” towards making the unconscious readable (1900/1942)—, but first and foremost because the key target of psychoanalysis are the signifiers produced by patients (‘analysants’) in their efforts to articulate their symptoms, their inhibitions, their anxieties, their desire.

For Lacan, linguistics is crucially important because it allows him to address a number of fatal misunderstandings which infected psychoanalytic discourse, notably concerning the nature of the unconscious and the position of the subject. The unconscious is *not* the seat of (pre-linguistic, untamed, animalistic, egoistic) “instincts” (1966a, p. 495), but rather structured like a language: the “discourse of the Other” (p. 524), preceding and predetermining the subject (p. 495), so that the latter is not the autonomous, rational, self-realising, choosing entity of ego psychology (and of behavioural economics), trying to ward off the unconscious instincts with the help of various mechanisms of defence. Rather, the subject is a product of the elementary structures and permutations of language.

Let me elucidate this with the help of a classic example from ethical discourse, the case of Antigone, the heroine of the Greek tragedy written by Sophocles (around 441 B.C.) which explicitly stages an (unsolvable?) conflict between two types of normative claims, referred to by Hegel (1807/1986, p. 322, p. 348, 1821/1970, p. 257, p. 294) as human and divine Law. In contrast to the former, the latter is unwritten and eternal and instead of questioning its legitimacy (which would already represent an act of infidelity) subjects are expected to stubbornly persevere (“verharren”; Hegel 1807/1986, p. 322) in their loyalty to the divine Law at all costs. From a Lacanian perspective, Antigone is not driven by a kind of sororal clan instinct, set into motion by the disgusting image of her brother’s unburied corpse, left behind on the battlefield, but rather by her conscience, that is: by divine Law, by the voice of the Other; a demonic incitement, imposing itself on her, coming from ‘elsewhere’ (i.e. the unconscious), clad in the linguistic form of an *unwritten* law and fuelling an inescapable clash with the Theban authorities and their *written* laws. In fact, Sophocles’ *Antigone* is the text where the signifier ‘autonomy’ (αὑτ ό νομος) is used for the very first time (Zwart 1993). From a Lacanian perspective, however, it is clear that she is not making ‘her own free choice’ (in the modern sense of ‘self-determination’). Rather, her fatal (and extremely unprofitable) craving to adhere to this unwritten law is imposed on her by an ‘extimate’ calling, as Lacan would phrase it: both *intimate* (coming from within, and therefore un-ignorable) and *external* (coming from ‘elsewhere’). It is a silent voice, which nonetheless cries out, a decisive instigation, resulting in her fatal *passage à l’acte*.

For contemporary readers, it has become difficult to really experience the excessive, demonic nature of Antigone’s divine calling because the signifier *autonomy* has been affected by a series of rather drastic displacements. If we follow its trajectory diachronically (through history) it is clear that nowadays the term no longer refers to the same ‘signified’ as in ancient Greece (Zwart 1993). In Kant, for instance, ‘autonomy’ means acting in accordance with the law of reason (as a rational agent), which is quite unlike the opaque, unfathomable (‘irrational’, heathenish) divine injunction to which Antigone commits herself. And in contemporary morality, ‘autonomy’ has acquired a rather liberal meaning, something like: acting in accordance with one’s own predilections. Nonetheless, the ancient association with pre-modern ideas is unconsciously retained, so that we are less free (less modern) than we think; still driven by extimate, opaque injunctions (at times resurging as the return of the repressed).

## The primacy of the signifier: speaking subjects and speaking masses

Thus, for Lacan, the rereading of psychoanalysis with the help of De Saussure’s linguistics rehabilitates Freud’s original insights, distorted by later vulgarisations, notably the insight that the moral subject is not an autonomous ego, but rather a chronically divided entity, torn between incommensurable normative claims. On the one hand, the subject is faced with what De Saussure refers to as the “speaking masses” (p. 112), comparable to what Heidegger (1927/1986) refers to as *Das Gerede* or *Das Man*: the moral chorus, voicing the accepted (‘ratified’) precepts of morality, the generally acknowledged associations between signifier and signified, institutionalised as ‘human law’. On the other hand, there is a different chain of associations between signifier and signified, coming ‘from elsewhere’, as Antigone (the ancient Greek ‘analysant’) phrases it: another language, a different truth. And it is precisely *because* Kreon so rigorously forbids her to live up to her extimate calling, that the desire to act in accordance with her truth (captured by the inspiring signifier *autonomy*) becomes so irresistible.

De Saussure likewise pointed out that the dual nature of linguistic phenomena (as a series of associations between signifier and signified) corresponds to the duality of human personhood: with the ‘signifier’ representing the external dimension (vocal sounds, writing, etc.) and the ‘signified’ representing the internal (psychic) dimension (p. 145). And whereas speaking subjects dwell in language as an imposing collective *system* of associations, there is an “inner treasure” (171) of idiosyncratic associations which is individually unique and may be brought to the fore via (interminable) processes of working-through (such as psychoanalysis or autobiographic writing).

Thus, Lacan uses De Saussure to rectify, but also to *radicalise* and recast the Freudian heritage, but the reverse is also true: he uses Freud to recast and radicalise linguistics. For rather than merely treading in De Saussure’s footsteps, some displacements are effectuated as well, and by far the most noteworthy one involves the algorithm S/s as such. For whereas De Saussure placed the *signified* (s) on top of the bar and the *signifier* (S) underneath [s/S], Lacan reverses their positions [S/s]. Although this is not immediately *at odds* with De Saussure’s theory (because, according to the latter, their position, either above or below the bar, is arbitrary), it nonetheless rather outspokenly reveals Lacan’s intention of emphasising the autonomy, the primacy, the commanding authority, the ‘tyranny’ even of the signifier (in accordance with psychoanalytic experience), while at the same time underscoring the “heteronomy” (p. 524), the dependence, the thraldom of the moral subject vis-à-vis the symbolic order. Thus, Lacan advocates a ‘literalisation’ of the subject as a product of the ‘play of signifiers’, haunted by language, with the paradoxical result that in Lacanian psychoanalysis the (moral) subject is initially *eclipsed* (by the omnipresent power of structures and systems), but subsequently *reinstalled* (as the speaking subject who desperately takes the floor to articulate (in a frantic, polemical dialogue with the discourse of the Other) the call of unconscious desire tormenting him or her.

Besides the unconscious and the subject, Saussurean linguistics allows Lacan to clarify a third decisive dimension of psychoanalysis, and of bioethics, namely sexual difference. As pointed out, De Saussure sees language as a system of differences, exemplified by (the presence or absence of) specific components. From a Lacanian perspective, it is no coincidence that De Saussure uses *père* and *mère* to elucidate this because language is imbued by sexual difference, notably in the form of male and female (vs. neuter) nouns. Psychoanalytically speaking, the consonants *p* and *m* became associated with the presence or absence of a particular member (or *articulus*, as De Saussure phrases it) in *male* or *female* as signified concepts. This also highlights an importance difference between Lacan and Freud. For whereas Freud connects sexual phenomena surfacing in psychoanalytic practice (such as: castration anxiety, fetishism, penis envy and the like) with the absence or presence of physical, corporeal items (with ‘members’ in the organic, anatomical sense of the term), for Lacan the phallus is basically a signifier. In other words, Lacan takes the phallus *literally*, which may sound cryptic, but can easily be explained. For Lacan (1966a, b, p. 499), the perfect exemplification of the functioning of the phallus is the following:

It is the ‘authority’ of the signifier (*Men* vs. *Women*) which imposes “urinary gender segregation” (p. 500) on individuals who find themselves away from home (on airports or in cafés), even if no physical difference can be detected between the two separate rooms (where men and women are temporarily kept in isolation), nor between the two lavatories to which the twin doors provide access (as in principle, there is nothing definitely ‘male’ or ‘female’ to a standard WC). Still, the signifier clearly states that men ≠ women, separating them into two parallel “processions” (p. 500).

Subsequently, sexual segregation (imposed by the phallic signifier) may function as a model for other forms of differentiation, such as age (at what age is someone entitled to give or withhold consent?), or time schedules (when is it allowed to enter a certain building?), or nationality (when is a particular individual entitled to certain health care facilities?). In the latter case, for instance, nationality is a ‘literal’ issue, indicated by a signifier, present or absent in printed documents such as passports, connected to systems of signifiers, in short: the symbolical order, rather than to something physical (as nationality is not immediately discernible in someone’s ‘genetic passport’). Likewise, biomedical signifiers (indicators, markers) allow us to segregate the normal from the pathological.

Corporeal elements such as penises and breasts, due to their appendix-like form, in combination with the fact that they are present in some and absent in others (and therefore detachable to some extent from the body as a whole), may provide a starting point for differentiation. Indeed, the existence of sexual difference is (one of) the first (unsettling) discoveries made by very young children. Perhaps at a certain point in history there was a more direct link between a particular signifier (say, q or l) and a specific member in the corporeal sense, similar to how the letter A (once shaped as ∀, aleph) originally functioned as an pictogram of an ox. Still, Lacan emphatically endorses the Saussurean viewpoint that signifiers are *arbitrary* and in no way refer to (or resemble) real things.^3^ In other words, the signifier *produces* segregation, *produces* male and female subjects, as well as healthy citizens and psychiatric patients. But this is the signifier’s typical mode of operation: structuring the world by introducing dichotomies, also in the moral domain, between good versus evil, ethical versus unethical, legal versus illegal, compulsory versus voluntary, virtuous versus vicious, significant versus insignificant, absence versus presence (of informed consent forms or codicils for instance), etc.

Thus, ethics as a system of signifiers propagates dichotomisation, thereby introducing a semblance of order, although actually, as moral deliberation continues to proceed and moral culture continues to evolve, the anchoring points (*points de capiton*; Lacan 1966a, b, p. 503) between the terminological grid (the signifiers) and the floating mass of convictions and experiences (the signified) remain highly precarious. The signified will continue to evade the grasp of the signifier, so that eventually nothing stops the incessant sliding of meaning (Lacan 1966a, b, p. 502; Nancy and Lacoue-Labarthe 1973/1992). Indeed, as Nietzsche (1881/1969, p. 1012) phrased it, even to *ask* questions concerning established conceptions of good and evil inevitably amounts to *questioning* them, thereby, perhaps unwittingly, subverting or loosening the fragile linguistic rapport between S (the signifier) and s (the incessantly sliding mass of opinions and ideas).

In view of the primordial position of the ethical signifiers (as weighty words), one cannot afford to use them too loosely. Rather, they must be taken to the letter, and it is not allowed to replace a signifier like ‘autonomy’ by other terms which may *seem* to come quite close (such as ‘liberty’, ‘independency’, ‘emotional stability’, etc.). Although such displacements easily occur in every-day moral discussions, in ethics (due to the careful handiwork of professional ethicists) key signifiers have acquired a rather stringent meaning, in connection with other linguistic elements within the system, so that one should take care to use them literally. Furthermore, signifiers such as ‘autonomy’ or ‘justice’ are not to be mistaken for descriptions of actual states of affairs (‘autonomy’ as a characteristic of a particular human being), but rather as performative imperatives, summoning someone to *become* autonomous, or to *become committed to* justice, guiding subjects in this direction, but also operating as a constant reminder of our inevitable failure to really live up to these commanding expectations. And that is why we, as moral subjects, notwithstanding the warning uttered above, are always on the look-out for smoother substitutes, less burdened with etymological, cultural or moral heritages,—e.g. ‘self-determination’ as a (smoother, less commanding) alternative for ‘autonomy’.

Lacan connects displacement with ‘metonymy’, again a term adopted from linguistics. Displacement/metonymy may function as a mechanism of defence against the harsh censorship exercised by our ruthless conscience, operating as an over-demanding, hyper-critical, ‘extimate’ voice or gaze. Metonymy may blunt the signifier’s cutting edge and evade its suffocating grasp. Subjects may even become fixated on their terminological alternatives, allowing them to temporarily evade the disquieting truth conveyed by the original, harsher term (p. 518). Thus, metonymy becomes part of the symbolical rhetoric of bioethical discourse (Lundberg 2012). It is primarily by carefully choosing their terms, by preferring neutralising and politically correct euphemisms over provocative labels, or vice versa, for instance, that participants position themselves vis-à-vis the symbolical order: genetic modification or manipulation?; euthanasia or mercy killing?; GM crops or Frankenstein food?; animal testing or vivisection?; bioethical principles or Georgetown mantra?; etc. And yet, Lacan argues, linguistic engineering (and this includes the careful conceptual handiwork of academic ethics) will never put an end, once and for all, to the linguistic struggles and confusions holding sway in the *opera*-*buffa humaine* of moral or social life (p. 526). Even Stalin (1950) had to admit that, notwithstanding the disruptive transformations unleashed by the October revolution (the demolition of the pre-revolutionary cultural superstructure), the Russian language remained more or less the same, although some phrases were added and some words were dropped (Lacan 1966a, b, p. 496).

## The topology of the moral landscape (*I*, *S* and *R*)

Linguistics provides a ‘royal’ bridge between psychoanalysis and bioethics, allowing us to explore the relationship between the subject and the *system* of signifiers, the symbolical order. From a Lacanian perspective, however, this is only one of three dimensions of the moral landscape, namely the *Imaginary*, the *Symbolical* and the *Real* (ISR).

I already briefly referred to these dimensions above. Initially, we respond to provocative images, such as the claustrophobic façade of the psychiatric building or the intimidating Gestalt of the Nurse, and the various phantasies and scripts to which they give rise. But before long, the ensuing moral discussion becomes increasingly verbal, with the help of terms and phrases, although such deliberations (either spontaneous or formalised) will prove unable to address the case to the full, due to a stubborn, annoying, inarticulate remainder: the ungraspable Real. Together, *I*, *S* and *R* constitute the ‘topology’ of the moral landscape.

The *Imaginary* may refer to pronounced examples of wrongfulness, or to utopian visions of healthy human bodies flourishing in peaceful societies, as a source of inspiration. Lacan (1955–1956/1981) mentions ancient statues of Greek and Roman athletes erected for the purpose of moral propaganda, but one could also think of Soviet posters depicting vigorous men and women of the future, or commercial advertisements depicting superbly healthy individuals who used the right diet or just recovered from a successful kidney transplant. Billboards displaying happy organ recipients in pro-donation campaigns (or transgender mannequins in fashion magazines) may be highly seductive, convincing enough us to make us enlist as donors for instance, but they may also raise suspicion: will organ transplantation (or transgender surgery) really achieve full recovery of the recipient’s integrity and health?

By asking such questions, we already enter the symbolical order, the grid of operational signifiers, of weighty, value-laden terms and imperatives, of operative guiding principles and the regulations build around them: in other words, everything Hegel refers to as *Sittlichkeit*. From a Lacanian point of view, bioethics as a discourse reflects the primacy of the signifier (S) vis-à-vis the steady stream of everyday moral associations and considerations (s). It was only *after* the term ‘autonomy’ was established (to capture a particular set of ideas) that this concept could be systematically worked through. Or take Kant’s famous essay *What is Enlightenment?* in which he manages to make a diffuse idea discrete and precise, pointing out what Enlightenment (*Aufklärung*) really *is* (Kant 1784/1971). In other words, the signifier stabilises, but to a certain extent even *precedes* the actual meaning of the term. Kant’s *Critique of Practical Reasoning* is an extremely unpractical work; focussing solely on ethical grammar (the operative force of key terms such as *ought* and *should*), far removed from the fuzzy, diffuse casuistry of everyday existence. Nonetheless, such normative terms, once established, may structure moral situations, acting as vectors, putting us on the track of interpretations and interventions. The signifier ‘injustice’ basically means that something should be done about the situation.

Thirdly, there is the dimension of the Real (not to be confused with reality: i.e. that which is actually perceived and experienced, a particular moral problem situation for instance). The Real is that which persistently resists symbolisation, remains impossible to articulate, revealing itself quite unexpectedly, in the form of meaningless, pointless disasters for instance (such as a sudden lethal illness, caused by a genetic defect), but also tragic conflicts exemplify the intrusion of the Real, disrupting the smooth discursive process. The basic objective of symbolisation is to put an end to moral ambiguity and to liberate moral subjects from the perplexities of incompatible moral claims, but (according to Lacan) this interminable process will never be completed. As soon as we reach consensus, new discordances and anomalies are bound to erupt. Still, although the symbolic order fails to resolve our problems and its guidance proves limited, we cannot do without. Should the conviction that our daily activities are basically legitimate evaporate, moral paralysis would immediately set in. Even criminal, violent acts need justification: an areole of moral words and phrases.

## The fourfold of bioethical discourse

Lacan’s final step is to radicalise the process of symbolisation through *formalisation*: i.e. the effort to capture key concepts in mathematical symbols and algorithms, known as mathemes. A first example was already discussed above, namely the algorithm of signification (S/s). While the dimensions *I*, *S* and *R* constitute his topology, the mathemes (and the symbolical elements from which they are composed) constitute Lacan’s algebra (Assoun 2003, p. 61).

Lacan’s starting point is the divided (craving) subject, exposed to (haunted by) conflicting yet imposing demands; in Lacanian algebra: *$*. In sharp contrast to neo-liberal trends in current bioethical discourse, the Lacanian subject is not at all the autonomous, choosing, self-conscious Self, bent on self-realisation. Quite the contrary, *$* is paralysed by uncertainties, pestered by a sense of deficiency and impotence, suffering from a basic inability or lack (in Lacanian algebra: −φ), always on the look-out for something which may relieve his/her problems by restoring a sense of competence and wholeness. Lacan refers to this inexorable, missing, life-saving ‘something’ as the object *a*, the object-cause of desire: that which will allegedly suture the gap or lack (in algebraic symbols: *a*/−φ).

The relationship between ‘subject’ (*$*) and ‘object’ (*a*) can be captured with the help of the so-called matheme of desire: *$* ◊ *a*, where *$* and *a* are separated/connected by a lozenge, an operator pointing in both directions, thereby indicating that the (impossible) object *a* is both the target and the cause of human craving. In bioethics, this missing object (*a*) may fuel the quest for (questionable) substitutes, such as the panacea (the golden bullet, the perfect prosthesis, the wonder drug, the unique ingredient, the compatible organ, purporting to make us whole again) or the perfectly implantable and/or wearable gadget, the smooth enhancer, allowing craving subjects to flourish once again, to overcome their short-comings and to live up to social (notably professional and sexual) expectations.

Two other important algebraic symbols are S_1_ (the Master signifier, the authoritative, imposing word of the Other) and S_2_: the chain of signifiers, in other words knowledge, although S_2_ may also refer to the expert, the custodian or spokesperson of this knowledge. With the help of this set of symbols (S_1_, S_2_, *$* and *a*), Lacan constructs four schematic structures, representing four basic types of discourse (Lacan 1969–1970/1991), the “summary and summit” of his oeuvre (Verhaeghe 2001). The four basic symbols (variables) may be inserted (in a fixed sequence: *$*, S_1_, S_2_ and *a*) in one of the four positions in a rotating, revolving, quadruped scheme:

In this manner, four discourses are generated.

The *Master’s discourse* is oriented towards the teachings of the Master (S_1_ as *agent*), whose name provides a guarantee of truth (such as, for instance, Hippocrates or Aristotle). The phrase *Aristotle dixit* (‘Aristotle said’) functions as truth certificate, adding weight to insights or statements attributed to this privileged source. The starting point (the speaking *agent*) is the Master, producer of the imposing signifier, the word of the Other (S_1_),^4^ addressing the scholarly reader (the philological expert, the curator of the Master’s intellectual heritage) as ‘other’ (S_2_ in the upper-right position). The repressed *truth* (lower-left position) of this type of discourse is the awareness that the Master surely must have been a doubting and divided subject himself (*$*), a dimension which is obfuscated, that is: hidden underneath (at the reverse side of) the bar. Although the experts seem to function merely as servants, the Master (like in the case of Hegel’s dialectics) becomes increasingly dependent on their labour, and the authoritative insights (S_1_) can only maintain their commanding presence because they are constantly commemorated, re-polished and updated by the servants, whose discursive dexterity continues to increase, so that in the end the Master’s discourse completely relies on their scholarly activities for survival, while eventually it is the scholarly expert who will determine what the Master actually said (distinguishing between what is to be maintained, underscored or ignored):

In contemporary academia, the contours of the Master’s discourse may be discerned in the philosophical genre known as ‘author studies’; devoted to actualising and vindicating the writings of authoritative authors such as Hippocrates or Aristotle. Initially, the Master signifier seems to give rise to a servile (philological, apologetic) discourse, wholly oriented toward explaining, elucidating and venerating the textual fragments and sayings of the Other. Yet, although this type of discourse may seem tedious, unprofitable and of limited societal relevance to outsiders, it produces a singular form of jouissance (*a*), thus providing an intellectual bonus, only accessible for those who really indulge in it. Moreover, unexpectedly perhaps, valuable insights, even for the present, may come from this committed return to the authoritative beginning, this fidelity to the original truth event: the untainted articulation of the inaugural Word.

The second type of discourse is *University discourse*, a logical next step, in view of the increased autonomy and power of the servant, pointed out above. Now, the starting point (*agent*) is the expert (S_2_) who, allegedly, refuses to accept any other authority besides scientific evidence and rational argumentation. In other words, the (unconscious, inspirational, authoritative) voice of the Other (S_1_), summoning the plodding, servile subject to produce more knowledge, is repressed, concealed or hidden from view (removed to the reverse side of the bar). It also means that (servile) reading practices give way to experimentation, by experts who take the process of truth production literally into their own autonomous, dexterous hands. Typically, university experts tend to devote their whole professional career to unravelling one particular privileged ‘object’ (be it a virus, a molecule, an elementary particle, a mathematical problem, a work of art or, in the case of bioethics, a particular ethical concept or problem, although by way of displacement the expert may also become fixated on a derivative objective, a citation index (*h*-score) for instance, or some other ‘perverse incentive’ at work in the current research arena. Such objects absorb all of the expert’s energy and time, at the expense of everything else, in the hope that, in the end, truth may be revealed, or academic prestige may finally be granted. But often, this unique target of the researcher’s *cupido sciendi* (the subject’s Will to Know) will prove a toxic, addictive lure and the unforeseen side-effect of this discourse often is a deadlock of personal malaise (*$*), so that the individual researcher actually becomes a victim of science, due to lack of response and acknowledgement, or because the object proves an evaporating fiction, or because the effort simply cannot be sustained. A classic example of course is Faust who, at the height of a promising scholarly career, suddenly succumbs to a mid-life crisis, so that his pupil Wagner continues and completes his work, but Lacan mentions a whole series of victims of science, including what seem to be highly successful researchers and heroes of science (J.R. Mayer, Boltzmann, Cantor, etc.: Lacan 1966b, p. 870). Biographies of famous scientists, allegedly success stories, are often tainted by severe experiences of failure, despair and fraud; the history of science as *hystory* (Verhaeghe 2001, p. 30):

This brings us to the third type of discourse (the third discursive ‘quadruped’), namely the *hysteric’s discourse* (or *hysterical discourse*), although this label should not be taken in a pejorative sense. Now, the divided, craving subject (*$*) vehemently takes the floor, raising a voice of protest (Stop scientific misconduct! Power to the patient! Self-determination!) to critically address the Master: an authoritative figure on duty, a representative of established discourse, whose system of signifiers is criticised and questioned. What is repressed/obfuscated is the object *a*, the true cause of the subject’s desire, fuelling the subject’s (often misdirected and therefore insatiable) manifest critique. This genre of discourse figures prominently in societal debates on technological issues (such as, for instance, genetic modification). As an unexpected product (side-effect), however, new questions may be asked, new avenues for research may be opened up, so that new insights may be generated (S_2_ in the lower-right position):

A strength of the hysteric’s discourse often is its acute seismographic sensitivity towards the undercurrents of social life, about to erupt. The movie *One flew over the Cuckoo’s nest* adheres to this structure, as a supportive voice endorsing the anti-psychiatry movement (Cooper 1967). The question is, however, whether such a crusade against authority will prove a viable strategy. Its blind spot is a lack of awareness of what is really pushing individuals such as Randle McMurphy forward: the object-cause of their desire (their object *a*).

But to bring this relationship between *$* and *a* to the fore, we need to make another leap and enter a fourth discourse (or quadruped), namely the discourse of the *analyst*, a paradoxical term, since (ideally) the analyst is the one who does not speak, but rather listens, with evenly-poised attention. But precisely because of the analyst’s self-constraint, the floor is now open to the subject’s repressed desire, provoked by the object *a*: that which stalks the divided subject (*$* in the upper-right position, challenged by *a*). In order for this type of discourse to unfold, however, knowledge and expertise (S_2_) must be suspended (*Docta ignorantia*, learned ignorance). Rather than interpreting and analysing the subjects’ problems, the analyst must invite the actors themselves to discover the cause of their malaise, the content of their true desire. Historically, this type of discourse was inaugurated by Socrates. As an unexpected side-effect, however, the analyst (Socrates, Freud, Lacan, etc.) may unwillingly be placed in the position of the Master (S_1_ in the lower-right position), producing new instances of venerating serfdom (as in the case of the Freudian movement, for instance, often criticised for systematically imbuing conformism and ostracising ‘deviationism’):

Bioethics may assume any of these four roles and, depending on time and context, its discursive profile may significantly shift. Jan Helge Solbakk, for instance, in the essay which was cited in the beginning of this paper, argues that, whereas academic bioethics in its infancy “lent its ear to the silenced voices in our societies”, today’s bioethics “is using its intellectual and moral skills to serve the interests of the most powerful voices in our societies” (Solbakk 2013, p. 320). Instead of “speaking truth to power”, it has become “a handmaiden within the medico-industrial complex, a governance tool”. In other words, according to Solbakk, the structure of bioethical discourse has been reversed, from the discourse of the *hysteric* (advocating issues, speaking truth to power) into *university* discourse, so that the agent’s ethical *expertise* (once a mere by-product) is now prominently on display, with experts presenting themselves as the ones who are supposed to know. Bioethicists can be consulted as articulate, acknowledged experts (S_2_) who have achieved fluent mastery of the official ethical vernacular, helping lay persons to distinguish between valid and invalid arguments, between proper (ratified) and improper terms (the latter should be replaced via metonymy or banned).

Bioethicists may also continue to play the role of advocate, however, committed to a particular, singular cause, so that bioethics reflects the structure of hysterical discourse, a symptom of which is the predilection for theatrical gestures, for ‘acting out’, emphatically seeking media attention, so as to put neglected or obfuscated issues on the agenda. Hysterical discourse definitely aims to enter the Master’s field of vision and is bent on catching the eye and ear of powerful players. It is the discourse of the whistle blower, of advocates of patient rights, calling for liberation, sometimes using their own physical body as a screen or text. Animal activists, for instance, in their protests against the use of laboratory animals in biomedical research, may resort to corporeal gestures, chain themselves physically to a research facility’s fence, raise their voice with the help of graffiti, spread noisy messages via megaphones or social media, or paint signifiers (as exclamation marks) on their naked skin, thus calling attention to (what they regard as) unheeded suffering and obfuscated injustice: the adolescent stage, perhaps, of a moral debate which, gradually, may become more manageable (once the “native hue of resolution is sicklied o’er with the pale cast of thought”). The hysteric’s discourse is part of a long tradition of popular and intellectual protest, relying on provocative gestures and interventions, making full use of ‘body language’, nowadays in vogue in movements such as Femen, but actually going back (diachronically speaking) to the ancient cynics: a bold, impertinent, popular, gay, practical, provocative, theatrical, grotesque and decidedly non-academic form of moral critique (Sloterdijk 1983). In the end, however, the hysteric subject not only challenges the Master, but also depends on him, demands his presence. See for instance Lacan’s famous response to the hysteria of the 1968 student uprising, arguing that these students were actually longing for an omnipotent master: the obfuscated desire of Maoist protest (1969–1970/1991).

From a Lacanian perspective, however, bioethics eventually should aspire to play the role of ‘analyst’, heeding the discourse of various speaking subjects with evenly-poised attention, waiting for symptoms and signifiers to surface, as basic constituents for a diagnostics of the present. The analyst’s discourse builds on the insight that speaking subjects, occupying the upper-left position, as Masters (S_1_), experts (S_2_) or hysterics (*$*), only apparently act as agents: they *are* spoken and driven by desire, by a truth unknown to themselves (Verhaeghe 2001). From the analyst’s perspective, moreover, even the (apparently ‘negative’) figure of the hysteric actually plays a positive role, revealing gaps in established discourse, highlighting blind spots or deliberative routines which rightfully invoke objections, because something of importance has been forgotten or eclipsed, something of value which now has become impossible to articulate. And in the case of university discourse, the analyst focusses on symptoms of professional uncertainty, ambivalence and unease, camouflaged by the expert’s apparent fluency and subtlety: *Mind the gap!* (Verhaeghe 2001). In other words: the analyst as a rhetorician, an expert in the dynamics and modes of discourse (Lundberg 2012; Lacan 1977–1978, p. 4).

Interestingly, Lacan’s four discourses overlap to some extent with the four roles for bioethicists (or, more generally: for academics involved in Ethical, Legal and Social Aspects research) as described by sociologist Wieser (2011) on the basis of empirical research, namely: *scholars* (analysing debates from an academic distance); *collaborators* (presenting themselves as expert partners in interdisciplinary research); *advocates* (emphatically taking a position in the debate); and *facilitators* (creating a social space for dialogue). In these four roles, the contours of the Master’s discourse, the university discourse, the hysteric’s discourse and the analyst’s discourse can easily be discerned.

This does not imply that bioethics should strive to coincide with the analyst’s discourse completely and continuously. Quite the contrary, the analyst’s discourse would become shallow and empty if bereft of experiences (‘input’) gained from the other three discourses. Bioethicists may play various roles within the ethical spiral or cycle, leaping stepwise from the Master’s discourse (for instance: introducing students to the Hippocratic oath) to the university discourse (presenting bioethics as a specialised form of expertise) to hysterical discourse (calling attention to dysfunctional, obscene gaps) up to the analyst’s discourse (reading the symptoms, discerning the obfuscated truth in the folds and margins of the mainstream debate) and from there back to the first position: rereading master-thinkers such as Aristotle, Kant, Hegel or Heidegger, to reclaim forgotten signifiers that may allow us to strengthen our sensitivity and articulacy. I will now further elucidate these four discourses (these four discursive quadrupeds) with the help of two bioethical ‘files’, namely human reproduction and the research animal debate.

## First exemplification: human reproduction

My first exemplification concerns a bioethical arena which is actually quite close to psychoanalysis’s own preoccupations, namely human reproductive technologies. Freud (1898/1952) himself devoted one of his most bioethical papers to this topic, published in the *Wiener Klinische Rundschau*, a scholarly magazine for medical practitioners, arguing that professional physicians have an ethical duty to take an enlightened stance towards sexuality as a major causal factor in psychic malaise, although this undoubtedly may bring them into conflict with traditional morality. Freud here speaks as an expert, discussing causes and treatments of ailments (such as hysteria and neurasthenia) in clinical, professional terms, claiming expertise (S_2_ in the upper-left position as agent), supported by an enlightened world-view (S_1_ in the lower left position) and expressing the hope that one day, biomedical science will triumphantly take control over sperm and ova (as elusive objects *a*) by developing contraceptives, thereby effectively separating sexuality from reproduction, furthering sexual emancipation and reducing psychic malaise (p. 507). In other words: Freud (on this occasion) primarily represents the type of discourse referred to by Lacan as university discourse:

However, as the anticipated triumph over natural procreation gradually shifted “from utopia to science” (Zwart 2009), psychoanalytic discourse now proliferates in more than one direction. University discourse continues, although Freud’s blatant optimism is increasingly counter-acted by voices that highlight possible mental downsides of a scientific take-over of reproduction (*$* in the lower-right position).

Yet, other sections of the “psychoanalytic establishment” (especially in countries such as France) rather opt for a return to a Master’s discourse (Žižek 2006/2009; Perelson 2013), arguing that in permissive, post-oedipal societies the technification of human reproduction by biomedical experts will disrupt the basic ethico-symbolic coordinates of civilisation. This type of response culminates in a plea for a massive return to symbolic authority and oedipal order (S_1_ in the upper-left position) to halt our sliding toward global chaos, autistic closure and pathological narcissism (Žižek 2006/2009, p. 297).

Such a plea against biomedical power may easily evolve into a provocative and hysterical form of protest (*$* in the upper-left position of agent), challenging the authority of the technocrats (S_2_, unaware of the dangerous desire that actually fuels their policies: *a* in the lower-left position), and raising a “warning call” against looming Frankensteinian or Brave New World scenarios (Vacquin 2002), framing artificial reproduction as a major disruptive threat to human dignity, well-being and culture. Interestingly, this type of psychoanalytic agitation often focusses, not only on the imaginary dimension (the uncanny Frankensteinian image of the frozen embryo) but also on the linguistic dimension, contesting the systematic neutralisation, desexualisation and de-differentiation of language which gives rise to a “lugubrious discourse” (Vacquin and Winter 2013, p. 87) of political correctness, a soft version of Orwellian newspeak: an assault on language and its deep ontological truths (p. 88), taking us from ‘sex without children’ (the 1970s) via ‘children without sex’ (the 1980s) towards a full neutralisation of sexual difference (p. 86). Thus, the hysterical approach frantically endorses a re-sexualisation of desire and reproduction in terms of *père* and *mère*. Notwithstanding its vehemence, however, this discourse does give rise to provocative insights, such as the observation that the new technocratic, gender-neutral language entails some paradoxical results: “How could we move from the issues of the 1970s, the maximum of sexuality with the minimum of reproduction, to the exact opposite, the maximum of reproduction with the minimum of sexuality?” (Vacquin 2002, p. 28); Or: “At the beginning of the century, people were thinking about freedom through sexuality, at the end of the century, they wanted freedom from sexuality… Why?” (p. 29). What is the desire that actually fuels the technification of human reproduction?

Via such questions, discourse inevitably slides into a more analytic mode, already voiced by Lacan, who points out (while discussing a case of post mortem sperm donation) how artificial reproduction (and its discontents) allows us to pinpoint the symbolical nature of fatherhood, as fatherhood is now *literally* reduced to its quintessence: the name-of-the-father, especially in cases where the ideal donor apparently is a father of name and fame (preferably dead), a pure signifier: a label on a sperm sample whose surplus value stems from the genius’s surname (Lacan 1966a, b, p. 813, 1956–1957/1994, p. 375). At the same time, this prominent father is bereft of something, namely authoritative parental say in the child’s upbringing. In other words, now that these possibilities are no longer science fiction, they reveal the opacity of the quintessential “factor x” of fatherhood, confronting us with fundamental uncertainties concerning our basic signifiers, urging us to pose some key questions anew, such as: what is fatherhood, what is nature, what is technology and, eventually, why do we want this?

## Second exemplification: experimentation with animals

And first glance, hysterical discourse seems an obvious starting point for reconstructing the animal ethics debate, involving activists who vehemently call attention to instances of animal suffering. From a diachronic perspective, however, discontent in animal research actually began as unintended by-product of university discourse (*$* in the lower-right position: Zwart 2008, p. 104 ff.). In contrast to Descartes’ claim that animals (ontologically speaking) are basically machines, physiologists inevitably discovered that animals are sensitive organisms. Albrecht von Haller (1707–1777) for instance became a physiology expert (S_2_) by performing lengthy series of experiments on live animals in the early 1750s. By doing so, however, he discovered that Descartes was wrong. Animals are *not* machines. The (Real) properties of muscle tissue cannot be explained in a purely mechanistic manner, as it displays an intrinsic tendency to react when excitated (a phenomenon Von Haller referred to as ‘irritability’), thereby revealing a gap, a deficiency in the symbolic grid of Cartesianism. Yet, this important, polemical insight confronted him with an ethical dilemma. In order to understand animal life and to do away with authoritative philosophical claims (S_1_), such as the Cartesian (and Scholastic) idea that animals are machines, experiments had to be performed on live animals. But by doing so, the expert discovered that animals (notably mammals) are sensitive living beings, quite capable of experiencing pain and distress, thus recasting animal experiments as instances of torture. In others words, in order to uncover the “animality” of animals, vivisection was both necessary (from a methodological point of view) *and* repulsive (from an ethical point of view). Initially Von Haller argued that, although he found performing experiments on animals revolting, in the interests of truth this “cruelty” could not be avoided (Guerrini 2003, p. 65). Yet, vivisection increasingly began to trouble his sensitive mind. Finally, unable to solve his problem (*$*), he decided to leave the field of physiology altogether and to devote himself to theology, botany and verse-writing instead.

Or take the case of Johannes Peter Müller (1801–1858), the most prominent German physiologist of his generation (S_2_), who faced similar dilemmas and tried to alleviate the problem by performing his experiments on frogs rather than on dogs (which required considerable surgical dexterity), or by using anaesthetics (ether or morphine) to mitigate the research animal’s pain (Guerrini 2003, p. 78). Anaesthesia eliminated some major objections to vivisection, but precisely *because* it made vivisection less disagreeable, it also resulted in an increase in the number of experiments performed. Moreover, Müller clearly recognised the methodological drawbacks involved. A benumbed animal is no longer a normal, reliable model. In other words, although laudable from an ethical point of view, the use of anaesthetics was highly problematic from a methodological standpoint, so that the expert was torn between incommensurable normative claims: methodology versus ethics (Zwart 2008, p. 107). Eventually, unable to solve his dilemma in a satisfactory manner, he left the field, and his moral malaise (*$*) may even have contributed to his suicide (although this is still a controversial issue among historians).

These experiences can be captured with the help of Lacan’s quadrupeds. Initially, the scholarly view of what animals (ontologically speaking) *are* was pre-structured by a Master’s discourse. Thomas Aquinas already argued that animals are basically machines (Zwart 1997) and in the early modern period, Descartes became an authoritative voice, as we have seen:

Discontent in this type of theoretical discourse, however, initiated a more experimental and scientific (hands-on) approach, provoking an epistemic rupture and giving rise to physiology as a research field. A particular form of expertise (S_2_) now functioned as ‘agent’ and discourse took a quarter turn to the left:

The authoritative voice of the Other is ignored (‘repressed’), although subconsciously S_1_ remains functional insofar as physiologists engage in a tacit polemics with this discourse of the Other. Thus we witness the social production of a new form of expertise, which initially was barred or discouraged by the Master’s discourse. That which was left out of the picture: the recalcitrant, inarticulate Real of Cartesianism, now becomes physiology’s research target of choice (*a*), to which researchers devote their career: the sensitivity (‘irritability’) of the living organism, to be explored experimentally. But this again produces a situation of malaise, as we have seen, so that the experts become the victims of their own research (*$*) and physiology becomes an impossible profession, questionable *from within*.

It is important to notice, by the way, that Descartes likewise worked as an empirical researcher for some time, conducting anatomical explorations as a polemical practice and as a route of escape from the authoritative voice of Scholasticism (*his* version of the discourse of the Master: S_1_), thereby paving the way for anatomy as a *university* *discourse* (S_2_). In the eighteenth century, however, Descartes (ironically, perhaps) had become an authoritative voice himself (S_1_ in the upper-left position), giving rise to a new polemical practice, namely physiology, resulting in a new exemplification of what Lacan refers to as *university discourse*.

Gradually, however, due to technologies of ‘refinement’ (S_2_), experimentation with animal models became less revolting, so that the *real* suffering of animals was partly reduced, and partly obfuscated. As a result, in the course of the twentieth century, the practice of animal experimentation became morally more sustainable, so that the number of animal experiments increased exponentially. The problem of professional malaise (*$*), which had threatened to turn animal physiology into an impossible profession, was reduced or by-passed, but never completely abolished, so that in the 1970 s (the era of anti-psychiatry, but also of a new wave of anti-vivisectionism) this obfuscated truth was exposed by critical voices *from outside*, by advocates and activists, acting in accordance with the structure of the hysteric’s discourse.

In this new discourse, the official, institutionalised view (S_1_ in the upper-right position) is challenged, namely that animal suffering is taken care of by trustworthy biomedical researchers who, in partnership with professional bioethicists, developed the necessary expertise, as a by-product of animal research (S_2_), acting as participants in animal ethics committees, carefully and conscientiously balancing animal ‘discomfort’ against societal relevance, and committed to reducing suffering via technical ‘refinements’. The hysterical voice which challenges this position is regarded as unreasonable/emotional (*$*) by the establishment, and as inspired by latent motives, such as the joy of rioting, or of enacting anarchistic or anachronistic (technophobic) ideas. Nonetheless, the by-product of this clash may consist in a broadening (enrichment) of the ethical vocabulary, or in a quest for reliable alternatives, as substitutes for in vivo animal models, such as in silico models (computer programs) or cell cultures (S_2_, now in the lower right position, as by-product of the debate):

But such a reframing of the animal ethics debate in terms of conflicting discursive structures requires another perspective, quite unlike the other three, namely the *analyst’s* discourse, revealing what makes these other discourses so tempting at first, but also how each of them leads into a deadlock sooner or later, so that speaking subjects seek solace in another type of discourse, shifting their position, taking a quarter turn, venturing a discursive leap:

From an analyst’s perspective, the strength of the Master’s discourse is that it challenges us to leave behind our daily, intuitive views and experiences (concerning animals for instance), and to adopt an (initially) quite startling, daring and productive view, in this case the idea that (human and animal) bodies are basically machines, an insight which is allegedly confirmed by anatomical data (S_2_).

Yet, sooner or later, in our interactions with real animals, notably in research practices that are more demanding and confronting, the gaps and shortcomings of this Master’s discourse, giving rise to all kinds of moral and epistemological anomalies, can no longer be denied, and subjects at a certain point are forced to take an *epistemological leap* from Master discourse (where, in the upper-right position, they act as recipients and custodians of the Master’s truth) to a different type of discourse, now occupying the upper-left position of agent themselves. And this is a liberating experience, for now, the reading of books (absorbing the voice of the Other) gives rise to something else, namely: real interaction with living organisms, so that subjects achieve autonomy by developing a methodology, a dexterity, a ‘body of knowledge’ of their own (S_2_).

But this new practice (notwithstanding the inviting avenues for research opened up and the intellectual jouissance it offers) eventually strands as well, as we have seen, so that once again new research practices have to be installed, allegedly less violent and revolting. And yet, again, there is an intrusion of the Real, as the problem of the physical and ontological violence implied in reducing animals to experimental materials and laboratory tools is obfuscated rather than solved. But this again requires a leap into a new type of discourse, now coming from outside, aimed at disclosing or even disrupting animal experimentation (‘vivisection’, performed by experts in laboratories, hidden from view) as a revolting practice.

Initially, this clash between discourses involves a polemic in the imaginary domain: a clash of images, with research laboratories publishing glossy pictures of perfectly healthy animals in annual reports, to which animal activists respond with shocking reproductions of sadistically tortured animals in leaflets, advertisements and brochures. Both types of images arouse suspicion, both are besides the truth, so that gradually, a symbolisation of the debate occurs, and the focus shifts from images to arguments, or even numbers (how many animals are used?; how much discomfort is inflicted?; how many therapeutic products are generated?, etc.). This shift towards a less image-driven debate also encourages the rise of a new type of university discourse, known as professional bioethics, enacted by ethical experts as participants in animal ethics committees.

Again, ideally, the bioethicist is someone who may play all these roles: reading the classics (S_1_), participating in ethical deliberations to coin or refine the signifiers (S_2_), but also raising a voice of protest when physical or ontological violence to animals is obfuscated (*$*). Precisely this alternating between various roles culminates in the oblique perspective of the analyst, revealing how and why this interminable (and in many ways repetitive) process (which unfolds both *within* and *between* discourses) continuous to spiral and evolve, so that, in the course of time, an incredible amount of knowledge (or at least discourse: books, articles, conference papers, etc.: S_2_) is produced, without ever coming to a stand-still, precisely because the truth of the matter will never be captured once and for all.

## Footnotes

1 In his dissertation Lacan analyses the case of Aimée, a psychiatric patient who, after writing two unsuccessful novels, stabbed a famous actress with a knife (Lacan 1932).

2 The word ‘term’ comes from the Latin *terminus*: i.e. the temporary endpoint of a diachronic process.

3 Unlike specific (iconic) symbols, which tend to retain some connection with tangible items (such as the balance, as a symbol for justice).

4 To paraphrase the Gospel of Saint John: *In principio erat verbum, et verbum erat apud Magistrum*.
